# Trace Elements in Medicinal Plants Traditionally Used in the Treatment of Diabetes—Do They Have a Role in the Claimed Therapeutic Effect?

**DOI:** 10.3390/foods11050667

**Published:** 2022-02-24

**Authors:** Pawel Konieczynski, Monika Gappa, Marek Wesolowski, Edgar Pinto, Agostinho Almeida

**Affiliations:** 1 Department of Analytical Chemistry, Medical University of Gdansk, Gen. J. Hallera 107, 80-416 Gdansk, Poland; monikagappa@gumed.edu.pl (M.G.); marek.wesolowski@gumed.edu.pl (M.W.); 2 LAQV/REQUIMTE, Department of Chemical Sciences, Faculty of Pharmacy, University of Porto, Rua de Jorge Viterbo Ferreira 228, 4050-313 Porto, Portugal; ecp@ess.ipp.pt; 3 Department of Environmental Health, School of Health, P.Porto, Rua Dr. António Bernardino de Almeida 400, 4200-072 Porto, Portugal

**Keywords:** antidiabetic plants, therapeutic effect, trace elements, ICP-MS analysis

## Abstract

Background: Medicinal plants are often used in the treatment of diabetes mellitus, although knowledge about their mode of action and the substances responsible for their antidiabetic potential is limited. It is well known that some trace elements play a role in glucose metabolism and insulin action. Thus, a particular trace elements profile could be associated with the antidiabetic properties observed for some medicinal plants. Methods: Infusions (*n* = 102) prepared from commercial herbal products (*n* = 34) containing medicinal plants indicated for the treatment of diabetes (*n* = 16 different plant species) and infusions (*n* = 60) prepared from commercial herbal products (*n* = 20) containing medicinal plants without such an indication (*n* = 7 different plant species) were analyzed by ICP-MS for their trace elements content. Results: In both groups, results varied significantly between different medicinal plants and also between different origins (brands) of the same medicinal plant. Significant differences (*p* < 0.05) between the two groups were found for nine elements, including four trace elements related to glucose metabolism (Mn, B, V, and Se), but with lower median contents in the group of medicinal plants for diabetes. Conclusions: Except for some particular species (e.g., *Myrtilli folium*) in which the trace element Mn may play a role in its antidiabetic effect, globally, a direct association between the claimed antidiabetic properties and a specific trace element profile of the studied medicinal plants was not evident.

## 1. Introduction

Diabetes mellitus is a group of chronic metabolic disorders characterized by hyperglycemia [1]. It is one of the main “civilization diseases” and represents an important burden for health systems worldwide, especially in more developed societies, as it is related to serious health problems [2].

Conventional pharmacological treatment to control blood glucose levels consists of two main groups of drugs—insulin and oral antidiabetics—depending on the underlying defect (inadequate insulin secretion or decreased tissue response to insulin) [3].

Medicinal plants have a long history of use [4] and, due to their generally ready availability, low cost, and minimal adverse effects, they continue to play a central role in the therapeutic arsenal of many populations, especially in the treatment of diabetes mellitus [5]. According to global ethnobotanical information, about 800 plant species are reported to be used in the treatment of diabetes mellitus, and for about 450 species there is experimental evidence of their antidiabetic properties. However, comprehensive knowledge of the mechanism of action has been obtained for just over a hundred of them [6]. Considering three types of evidence (“uses supported by clinical data”, “uses described in pharmacopeias and well-established documents”, and “uses described in traditional medicine”), several plants species are mentioned in WHO Monographs on Selected Medicinal Plants as having antidiabetic potential [7].

Several trace elements have been associated with glucose metabolism, mainly because of their role as cofactors of enzymes involved in glucose metabolism, their antioxidant properties, and their enhancement of insulin action through activation of insulin receptor sites [8,9]. Chromium (trivalent) has been shown to improve glucose metabolism in several animal and human clinical studies, including in diabetic patients [10]. Putative mechanisms by which Cr upregulates cellular glucose uptake include potentiation of insulin action, enhancement of the insulin signaling pathway, blunting of negative insulin signaling regulators, and enhancement of AMPK (AMP-activated protein kinase) activity [11]. Zinc is a key element in human metabolism due to its action as a cofactor for the functioning of many dozens of intracellular enzymes [9], playing an important role in β-cell function and insulin action [12]. It is involved in the stabilization of insulin hexamers and insulin storage in the pancreas and appears to have some insulin-like properties [8]. Additionally, it is an important antioxidant, a remarkable property in this context, since oxidative stress is linked to insulin resistance and the progression of diabetes [8]. Selenium is a key element in the defense against oxidative stress, and its antioxidant and anti-inflammatory effects may play an important role in preventing the development of diabetes and its complications [9]. Vanadium affects several signaling cascades in glucose metabolism, acting primarily as an insulin-mimetic agent [9,13]. Increased insulin activity and sensitivity have also been noted among its effects [9]. Copper has shown insulin-like activity, and Cu deficiency results in glucose intolerance and insulin resistance [9]. Manganese is required for the normal synthesis and secretion of insulin by pancreatic cells [9] and treatment with Mn has been shown to improve glucose tolerance under conditions of dietary stress [14]. Cobalt has also shown hypoglycemic properties, mainly due to its ability to decrease GLUT1 expression and inhibit gluconeogenesis [9]. In addition, there is some evidence that boron, also, plays a role in insulin and glucose metabolism [15].

This study aimed to assess whether trace elements can play a role in the hypoglycemic properties of medicinal plants traditionally used in the treatment of diabetes. For this purpose, the trace elements contents of infusions prepared from (i) medicinal plants traditionally used for the treatment of diabetes mellitus and (ii) other medicinal plants, without any reference to such use or antidiabetic properties, were compared. The main objective was to evaluate whether medicinal plants used for diabetes have a particular trace elements profile, different from other medicinal plants, which could somehow be related to their claimed antidiabetic effect.

## 2. Materials and Methods

### 2.1. Plant Materials

Plant materials were obtained as commercial products (*n* = 54) available at herbal stores. Two groups were considered:

(a) “*Medicinal plants used for diabetes”* group—consisting of 34 single plant products, corresponding to different brands of 16 medicinal plant species traditionally used in the treatment of diabetes mellitus. Seven of them corresponded to the medicinal plants with the best evidence of antidiabetic effect according to WHO monographs, i.e., plants with “uses supported by clinical data” or “uses described in pharmacopeias and well-established documents” [7].
(b) *“Other medicinal plants”* group—consisting of 20 single plant products, corresponding to different brands of seven species of medicinal plants without any reference to antidiabetic use or properties.

All products were purchased in Gdańsk, Poland, except for seven products with medicinal plants with indications for diabetes according to the WHO monographs, which were purchased in Porto, Portugal. Additional details of the brands (manufacturers) and geographic origin of the products are provided in the Supplementary Materials (Table S1).

Before analysis, the entire contents of a package were crushed in a knife mill (Retsch, Haan, Germany) and sieved (pore size 150 μm) to obtain a homogeneous material. The plant material was then stored in hermetically sealed polyethylene containers until analysis.

### 2.2. Infusion Preparation

Approximately 1 g of plant material was accurately weighed into a conical glass flask and 100 mL of boiling ultrapure water (obtained from an Arium^®^ pro water purification system; Sartorius, Goettingen, Germany) was added. Then, the flasks were covered with a watch glass and left at room temperature for 10 min. Approximately in the middle of this time period (~4.5 min), the contents of the flasks were shaken manually for 1 min. Thereafter, the infusions were centrifuged for 1 min at 4500 rpm (Heraeus Megafuge 16 Centrifuge, Thermo Fisher Scientific, Hemel Hempstead, UK) and vacuum-filtered using ashless filter paper (Whatman No. 42; GE Healthcare, Buckinghamshire, UK). Finally, about 50 mL of infusion was transferred to polypropylene tubes, 1 mL of high purity concentrated HNO_3_ (≥69% *w*/*w*, TraceSELECT^®^, Fluka, L’Isle d’Abeau Chesnes, France) was added, and the solutions were kept in the refrigerator until further analysis.

For each sample of plant material, three independent infusions were prepared. In total, 102 infusions were then prepared in the “medicinal plants used for diabetes” group and 60 infusions in the comparison group (“other medicinal plants”). Sample blanks were prepared following the same procedure.

### 2.3. Infusion Analysis

Multi-elemental analysis of the infusions was performed by inductively coupled plasma mass spectrometry (ICP-MS) using an iCAP™ Q instrument (Thermo Fisher Scientific, Bremen, Germany) operated under the conditions listed in the Supplementary Materials (Table S2).

The instrument was equipped with a MicroMist™ nebulizer (Glass Expansion, Port Melbourne, Australia), a Peltier-cooled baffled cyclonic spray chamber, a standard quartz torch, and nickel sample and skimmer cones. High-purity argon (99.9997%; supplied by Gasin II, Leça da Palmeira, Portugal) was used both as the nebulizer and plasma gas.

To avoid sample contamination, all labware was decontaminated by immersion for at least 24 h in a 10% HNO_3_ solution and rinsing with ultrapure water three times. All solutions were prepared with ultrapure water and high-purity concentrated HNO_3_ was always used to acidify (to 2% *v*/*v*) both samples and standard solutions.

Calibration standards were prepared by appropriately diluting two 10 mg/L multi-element standard solutions: PlasmaCAL SCP-33-MS (SCP Science, Baie-d’Urfé, Quebec, QC, Canada) and Periodic Table Mix 3 for ICP (TraceCERT^®^; from Fluka, Buchs, Switzerland). An internal standards solution (100 µg/L) was prepared by diluting three single-element standard solutions: Platinum Standard for ICP, Indium Standard for ICP, and Gallium Standard for AAS, all also from Fluka.

Prior to analysis, infusions were diluted 1 + 1 with 2% HNO_3_ and the internal standards solution was added. Elemental isotopes (*m*/*z* ratios) monitored in the ICP-MS analysis are shown in the Supplementary Materials (Table S3). The results obtained in the analysis of the infusions (μg/L) were converted and expressed as micrograms of element per gram of plant material (μg/g).

### 2.4. Analytical Method Validation

The limits of detection (LOD) were calculated as the concentration corresponding to 3.3× the standard deviation (SD) of 14 sample blanks and are presented in the Supplementary Materials (Table S3). To assess the accuracy and precision of the analytical procedure used in the infusions analysis, three types of experiments were performed: (1) direct analysis vs. analysis after acid digestion; (2) spike recovery experiments; and (3) analysis of a certified reference material (CRM).

In the first experiment, five randomly selected infusions were analyzed without any pretreatment (except the two-fold dilution with 2% HNO_3_) and after acid digestion in a microwave oven (Milestone, MLS-1200 mega, Sorisole, Italy). For the second experiment (spike recoveries), six infusions from two different plants were diluted two-fold, spiked with 1 and 2 µg/L multi-element standard solutions, and directly analyzed. In the third experiment, the Reference Material *Enviro*MAT™ ES-H-1 (Ground Water; from SCP Science) was analyzed under the same conditions as the infusion samples. The results of these experiments validated the procedure used.

### 2.5. Data Analysis

Statistical data analysis was performed using IBM SPSS Statistics 26.0 software (IBM Corp., Armonk, NY, USA). For mathematical calculations, the results below the LOD were imputed as the LOD divided by the square root of 2, a commonly used procedure to handle left-censored data [16]. The normality of variables distribution was evaluated by the Shapiro–Wilk test and the homogeneity of variances by Levene’s test. Differences in element contents between the two groups of medicinal plants were evaluated using the non-parametric Mann–Whitney U test. Statistical significance was considered for a *p*-value < 0.05.

## 3. Results

As detailed above, infusions prepared from two groups of medicinal plants were analyzed for their concentration regarding a wide panel of trace elements, including the most important essential trace elements and particularly those more closely associated with glucose metabolism (Mn, B, Zn, Cu, Cr, Mo, Co, V, Se). The first group (*n* = 102) consisted of infusions prepared from commercially available products containing medicinal plants indicated for the treatment of diabetes (“medicinal plants used for diabetes”; hereinafter “MP-for-diabetes” group). The second group consisted of infusions (*n* = 60) prepared from medicinal plants without any traditional or literature reference to antidiabetic properties or use in the treatment of diabetes (“other medicinal plants”; hereinafter “Other MP” group).

The results obtained for essential trace elements in medicinal plants (µg/g) as determined in their infusions (1 g sample: 100 mL boiling ultrapure water) are presented in Table 1, where they are detailed according to plant species. In many cases, three different brands (manufacturers) of the same plant species were analyzed (indicated by the digit after the dot in the product ID), but in some cases only one or two brands could be found commercially available. Products 1–34 corresponded to medicinal plants indicated for diabetes (“MP-for-diabetes” group). Of these, products 1–12 correspond to medicinal plants with a well-established therapeutic use in the treatment of diabetes according to WHO monographs (first and second categories, i.e., “uses supported by clinical data” and “uses described in pharmacopeias and well-established documents”). Products 35–54 were medicinal plants with other therapeutic indications (“Other MP” group).

In some cases, the elemental composition of the infusions was shown to be variable depending on the medicinal plant but also on the brand (supplier) analyzed. This is the case, for example, for *Salviae folium*, where Mn and Zn levels were significantly higher in infusions prepared from a particular brand (126.7 µg/g and 17.8 µg/g, for Mn and Zn, respectively), compared with the two other brands (43.0 and 40.6 µg/g for Mn; 9.2 and 8.7 µg/g for Zn). The same also happened with, for example, *Menthae piperitae* folium, whose Mn, Zn, and Cu levels were significantly higher in infusions prepared from a particular brand than in the other two.

*Medicinal plants used for diabetes versus other medicinal plants*

A summary of the results (µg/g) for essential trace elements according to the claimed therapeutic effect of the plants is presented in Table 2. Elements contents followed a quite similar order of appearance in the two groups of medicinal plants: Mn > B > Zn > Cu > Cr > Mo > Co > V > Se, in the “MP-for-diabetes” group and Mn > B > Zn > Cu > Cr > Mo > Se > V > Co in the “Other MP” group. The most abundant (median concentrations in the order of a few µg/g) were Mn, with a median content of 10.5 µg/g in “MP-for-diabetes” and 18.7 µg/g in “Other MP”, followed by B (11.7 µg/g vs. 16.0 µg/g), Zn (10.6 vs. 11.6 µg/g), and Cu (4.8 vs. 5.1 µg/g).

Statistical data analysis showed significant differences (*p* < 0.05) between the two groups for the mean values of Mn, B, V, and Se (Table 2), with lower values in the “Other MP” group, except for Mn. However, median content was lower in the “MP-for-diabetes” group for all of these elements. The overall mean content of Mn was highly affected by the large contribution of a single plant, *Myrtilli folium*. The Mn content in the infusions of this plant (598.2 µg/g and 498.8 µg/g) was much higher than in the others, both those used for diabetes and those not indicated for diabetes (Table 1).

*Medicinal plants used for diabetes covered by WHO monographs versus other medicinal plants*

Specifically considering only the subgroup of medicinal plants with a well-established therapeutic use in diabetes according to WHO monographs (the first and second categories, i.e., “uses supported by clinical data” and “uses described in pharmacopeias and well-established documents”), significant differences were observed in relation to the comparison group (“Other MP”) for Mn and B, with higher overall mean contents in infusions prepared from medicinal plants referred to in the WHO monographs for Mn and lower overall mean contents for B.

*Medicinal plants used for diabetes covered by WHO monographs versus other medicinal plants also used for diabetes*

Statistical data analysis also showed some significant differences between the subgroup of medicinal plants with well-established therapeutic uses in diabetes treatment according to WHO monographs and other medicinal plants also used for diabetes for four essential trace elements related to glucose metabolism, i.e., Mn, Cu, Se, and V, with higher contents in the subgroup of plants covered by the WHO monographs for Se and V.

*Toxic trace elements*

The results obtained for the main toxic trace elements determined (Ni, As, Pb, Cd, Tl, Sb, Be) are summarized in Table 3. All analyzed infusions showed detectable levels of Ni, Cd, Pb, and Tl. Other potentially toxic elements detected in most infusions included As (found in 60% of the infusions in the “MP-for-diabetes” group and all infusions in the “Other MP” group), Sb (found in 81% and 100% of the infusions, respectively), and Be (found in 62% and 95% of the infusions, respectively). The order of abundance was quite similar between both groups: Ni > As > Pb > Cd(Tl) > Tl(Cd) > Sb > Be. Details for the plant species and brands are presented in the Supplementary Materials (Table S4). A summary of the results obtained for a wide panel of other trace elements (not essential and not particularly toxic) is also presented in the Supplementary Materials (Table S5).

## 4. Discussion

Overall, the results obtained do not highlight a particular trace elements profile in medicinal plants used for diabetes.

There are different findings in other works, namely, some studies that showed significantly higher levels of Cr in medicinal plants used for diabetes [17,18]. In a comparative study by Castro [17] of Portuguese plants commonly used in folk medicine, significantly higher levels of Cr were obtained in those plants traditionally used for the treatment of diabetes (1.1–3.8 µg/g dw) than in other plants (0.62–1.2 µg/g dw). Very similar results were also obtained with Brazilian plants (1–4 µg/g dw vs. 0.5–1.5 µg/g dw, respectively) [18]. It should be noted, however, that these are total values (obtained after complete mineralization of the plant material) and not values for the infusions prepared with the plants, therefore not representing the actual intake by the people who consume them.

There are a number of factors that influence the elemental composition of medicinal plants. In addition to their intrinsic nature, namely, genetic aspects that dictate a different physiological behavior, trace element contents in plants can vary greatly depending on several factors, including geography-related conditions, such as soil composition and the climate of the region where they grew [19,20], as well as the age of the plant and the growing season.

Possible contamination must also be considered as a major factor that can cause inconsistent results regarding the trace element contents in plants of the same species but of different origins and this may explain the results obtained by us for the same plant acquired from different suppliers. Water and air pollution, cultivation in industrial and urban areas, agricultural practices, and the use of contaminated crop protection agents can also contribute to plants’ increased concentrations of various elements, both toxic (e.g., Cd, Pb, Ni) and essential (e.g., Zn, Cu), which can be transported directly from soil to the roots and accumulate in different parts of the plants.

Thus, increased levels of some trace elements in plants may reflect edaphic (soil) factors [21], but non-edaphic contamination can also significantly impact trace element levels in plants. This includes the contribution of foliar spray fertilizers and atmospheric precipitates, as well as dust deposits from the surrounding environment, which can easily be collected and adsorbed onto the plant surface [21].

External contamination of plants with soil dust must be considered one of the main contributors to the variability of the results obtained, especially for those elements that are very abundant in the earth’s crust. Thus, in particular, the content of elements, such as Al, Mn, Ba, Sr, Cr, V, Ni, Li, Cu, Ti, and Pb, in infusions may differ simply for this reason [22]. In addition, possible secondary contamination of plant material during harvesting and processing must be considered. Thus, the levels of Al, Cu, Pb, Cr, and Zn, for example, can increase when plant material comes into contact with metallic surfaces of equipment and machinery [21]. Contamination of plant material can still occur due to inadequate storage conditions.

On the other hand, there are also a number of factors that strongly affect the mineral concentration of medicinal plant infusions, naturally including the mineral composition of the plant (formulation), but also the pH of the water used for the preparation of the infusions, the preparation procedure itself, including the temperature and extraction time, the solubility of elemental compounds, and the composition of the vegetal matrix in particular components, which can condition the “extractability” of minerals [23]. A comprehensive compilation of literature data on the elemental content (macrominerals and trace elements) of medicinal plants and their tisanes, as well as the percentage of element release into infusions and decoctions, is provided by Pohl et al. [23]. For highly relevant elements, such as Cr, Mn, Zn and Cu, it has been shown that the “easily bioavailable fraction” can typically be less than 10% [24].

Although, globally, the highest overall mean contents of essential trace elements related to glucose metabolism have been found in infusions prepared from plants in the “Other MP” group (i.e., medicinal plants not used for diabetes), the highest levels of particular trace elements were found in some specific medicinal plants indicated for diabetes. This is the case already highlighted for Mn, whose highest levels were found in *Myrtilli folium* infusions (598.2 µg/g and 498.8 µg/g), followed by *Schisandrae fructus* (116.3 µg/g) and *Salviae folium* (126.7 µg/g in one brand; 40.6 and 43.0 µg/g in the other two) (Table 1).

Interestingly, the highest levels of Cr were also found in three plants in the “MP-for-diabetes” group: *Ginseng radix* (0.547 µg/g), *Schisandrae fructus* (0.448 µg/g), both medicinal plants with strong evidence of antidiabetic efficacy according to WHO monographs, and *Taraxaci radix* (0.552 µg/g; mean value for the three brands).

Similar findings were observed for B, with the highest contents being found in *Mori folium* infusions (35.7 µg/g; mean value for the three brands) and *Phaseoli pericarpium* (35.7 µg/g; just one brand), both plants in the “MP-for-diabetes” group.

Mo was highly variable between plants. The highest value was also found in infusions of a plant from the “MP-for-diabetes” group, *Astragali radix* (only one brand; 3.66 µg/g), followed by *Urticae folium* (“Other MP”; three brands; mean value: 0.93 µg/g) and *Trigonellae foenugraeci semen* (“MP-for-diabetes”; four brands, mean value: 0.78 µg/g).

Co was also higher in two plants from the “MP-for-diabetes” group, specifically two medicinal plants with strong evidence of antidiabetic efficacy according to WHO mono-graphs: *Trigonella foenugraeci* (0.182 µg/g; mean value for four brands), followed by *Ocimi sancti folium* (0.175 µg/g). *Ocimi sancti folium* infusions also showed a high level of V, significantly higher than the infusions from the other plants in the “MP-for-diabetes” group, but the highest value was found for *Chamomillae anthodium* (“Other MP” group): 0.264 µg/g (mean value for the three brands).

*Chamomillae anthodium* (“Other MP” group) clearly stood out. Zn levels were quite similar in all plant infusions, but the highest values (approximately 18–21 µg/g) were found for *Chamomillae anthodium* (followed by *Urticae folium*, also from the “Other MP” group). The highest Cu values were found in a plant from the “MP-for-diabetes” group, *Silybi mariani fructus* (three brands; mean value: 12.8 µg/g), followed by *Chamomillae anthodium* (“Other MP”; three brands; mean value: 12.2 µg/g). *Chamomillae anthodium* also presented the second highest levels of B (29.5 µg/g: mean value for the three brands) and relatively high levels of Co (0.127 µg/g: mean value for the three brands).

In any case, the contribution of the studied infusions to the daily intake of these elements did not prove to be very significant. For example, for Cr, the adequate intake (AI) for adult (19–50 years) men and women is 35 µg and 25 µg/day, respectively [25]. Even in the case where the highest values were obtained (approximately 0.5 µg/g), one dose (100 mL of infusion prepared with 1 g of the plant) would represent less than about 2% of the AI. The same goes for Mo, whose AI for adults (≥18 years), according to EFSA [26], is 65 µg/day. For the highest values obtained, an infusion dose (3.7 µg) would represent 5.7% of the AI, but for the median value (0.11 µg/g), an infusion dose would only represent about 0.17% of the AI.

The most significant exception was Mn in *Myrtilli folium*. The median value of approximately 10 µg/g obtained for medicinal plants used for diabetes treatment means that one infusion dose (100 mL) represents only about 0.3% of the AI for adults (≥18 years) of 3.0 mg/day [27]. However, for *Myrtilli folium,* it can reach approximately 20% of the daily AI.

Medicinal plant infusions can also be a source of potentially toxic elements. The levels of these elements also varied remarkably between different plants and, in some cases, between different brands of the same plant, but they were always quite low. However, when compared to drinking water quality standards, as defined in the Drinking Water Directive [28], the obtained results for the toxic elements covered by the directive (i.e., Sb, As, Cd, Pb, and Ni) were well below the parametric value in all analyzed infusions. Therefore, globally, there seems to be no reason for concern regarding the safety of the analyzed products. Among the other elements also analyzed (Supplementary Materials, Table S4). the presence of Sr and Rb naturally stood out: 11.4 vs. 15.4 µg/g (Sr) and 8.6 vs. 13.2 µg/g (Rb), for “MP-for-diabetes” and “Other-MP”, respectively.

## 5. Conclusions

The results from the multi-elemental analysis of infusions prepared from medicinal plants traditionally used for diabetes (1 g plant: 100 mL boiling ultrapure water) did not show a clear relationship between the claimed antidiabetic properties and a particularly rich profile of essential trace elements. In fact, no significant differences were observed compared to a control group (medicinal plants without any reference to antidiabetic properties or use) or their essential trace element levels were even lower. However, for some specific plants, the potential contribution of trace elements to their antidiabetic effects cannot be ruled out.

For the same plant species, the elemental composition of the infusions was sometimes somewhat variable, depending on the brand (supplier), which may reflect different growth conditions and exogenous contamination. That is, the determined trace element contents may reflect the external contamination of the plant more than the intrinsic contents related to its specific physiology, in which case they would be found not to be related to the antidiabetic efficacy of the medicinal plant.

To better clarify this issue, further studies should be carried out, promoting plant growth under equal and well-controlled conditions, or fresh plant material should at least be washed before the drying process to eliminate exogenous contamination.

The main limitation of this work is the rather small number of products studied. Since the content of trace elements in plants largely depends on numerous “extrinsic” factors (soil, environment, growing conditions, post-harvest processing, storage, etc.), it would be worthwhile to study more products, of more brands/from different manufacturers, and from a wider range of geographic locations.

The main strengths are that the most important medicinal plants with antidiabetic properties have been studied, namely, all those mentioned in the WHO monographs on Medicinal Plants as having the strongest evidence of those properties. In addition, they were compared with other medicinal plants for which these effects are not claimed. Furthermore, the comparison was made under exactly the same experimental conditions and the study focused on the contents of trace elements in infusions and not in the plant material itself, thus allowing to be taken into account the actual supply of trace elements to the human organism derived from the use of these plants.

Thus, it seems to be possible to conclude that the claimed antidiabetic effects of the plants studied do not depend on particularly rich contents of trace elements with a recognized action in glucose metabolism (the determination of which question being the aim of this study) but that these effects are mainly due, rather, to some organic compound(s).

In addition to contributing to the clarification of the mechanism of action of medicinal plants, this study provides current data on the contribution of their infusions to the recommended daily intake of trace elements (and evidenced a good safety profile of the tested products with regard to heavy metals contamination). It should be noted that the levels of trace elements in infusions of the studied plants can be much lower under real conditions, as their extraction was carried out with finely ground plant material, which naturally increases the yield of the extraction.

## Figures and Tables

**Table 1:** Levels (µg/g) of essential trace elements in medicinal plants as determined in their infusions (1 g sample: 100 mL boiling water).

Product No.	Plant. Brand	Plant Material, Plant Species, and Botanical Family	Cu	Mn	Zn	Se	Mo	Cr	V	B	Co
1	1.1	*Trigonellae foenugraeci semen, Trigonella foenum-graecum* L., *Fabaceae*	6.63 ±0.35	7.08 ±0.15	14.21 ±0.32	0.044 ±0.002	0.622 ±0.014	0.207 ±0.002	0.021 ±0.004	4.37 ±0.15	0.236 ±0.002
2	1.2		7.28 ±0.31	4.95 ±0.20	12.39 ±0.53	0.041 ±0.002	0.366 ±0.011	0.182 ±0.005	0.039 ±0.001	5.37 ±0.93	0.193 ±0.009
3	1.3		6.03 ±0.38	3.80 ±0.16	9.55 ±0.39	0.036 ±0.002	0.681 ±0.030	0.153 ±0.008	0.044 ±0.001	5.90 0.18	0.185 ±0.012
4	1.4		4.73 ±0.47	5.00 ±0.06	12.12 ±0.64	0.044 ±0.005	1.444 ±0.036	0.200 ±0.010	0.035 ±0.003	7.13 ±0.35	0.114 ±0.002
5	2.1	*Maydis stigma, Zea mays* L., *Poaceae (Gramineae)*	1.46 ±0.08	2.57 ±0.07	6.33 ±0.34	0.053 ±0.008	0.068 ±0.005	0.123 ±0.011	0.061 ±0.008	14.35 ±0.90	0.009 ±0.001
6	2.2		1.63 ±0.14	4.35 ±0.26	9.82 ±0.40	0.069 ±0.009	0.065 ±0.004	0.149 ±0.008	0.066 ±0.002	12.92 ±0.89	0.012 ±0.001
7	2.3		1.12 ±0.18	1.65 ±0.40	3.38 ±0.17	0.126 ±0.015	0.026 ±0.003	0.047 ±0.004	0.022 ±0.002	10.65 ±1.44	0.002 ±0.0003
8	3.1	*Ocimi sancti folium, Ocimum sanctum* L., *Lamiaceae*	4.42 ±0.39	12.97 ±0.71	5.50 ±0.46	0.116 ±0.007	0.036 ±0.002	0.212 ±0.010	0.150 ±0.007	16.52 ±0.47	0.175 ±0.006
9	4.1	*Azadirachti fructus, Azadirachta indica A. Juss., Meliaceae*	2.39 ±0.60	11.35 ±0.77	8.57 ±0.75	0.142 ±0.011	0.058 ±0.014	0.285 ±0.014	0.041 ±0.011	26.50 ±2.37	0.047 ±0.002
10	5.1	*Ginseng radix, Panax ginseng C.A. Meyer, Araliaceae*	6.00 ±0.99	28.27 ±1.07	11.75 ±0.72	nd	0.037 ±0.001	0.547 ±0.020	0.062 ±0.005	4.68 ±0.28	0.091 ±0.002
11	6.1	*Schisandrae fructus, Schisandra chinensis (Turcz.) Baill., Schisandraceae*	5.13 ±0.22	116.34 ±2.42	19.14 ±0.32	nd	0.051 ±0.003	0.448 ±0.011	0.111 ±0.001	12.30 ±0.24	0.069 ±0.001
12	7.1	*Astragali radix, Astragalus membranaceus (Fisch.) Bunge, Fabaceae*	5.32 ±0.35	6.59 ±0.15	8.43 ±0.29	nd	3.657 ±0.031	0.266 ±0.001	0.091 ±0.008	7.29 ±0.23	0.084 ±0.001
13	8.1	*Mori folium, Morus alba* L., *Moraceae*	3.46 ±0.26	5.75 ±0.42	14.9 ±0.61	0.023 ±0.007	0.257 ±0.013	0.215 ±0.014	0.027 ±0.001	33.70 ±0.86	0.022 ±0.0007
14	8.2		3.18 ±0.44	6.42 ±0.25	15.53 ±0.69	0.030 ±0.006	0.296 ±0.034	0.214 ±0.008	0.026 ±0.0003	34.12 ±1.09	0.022 ±0.0004
15	8.3		3.88 ±0.51	6.30 ±0.33	13.51 ±0.07	0.035 ±0.010	0.241 ±0.046	0.196 ±0.007	0.020 ±0.002	39.23 ±1.03	0.020 ±0.0004
16	9.1	*Phaseoli pericarpium, Phaseolus vulgaris* L., *Fabaceae*	4.94 ±0.65	11.11 ±0.17	8.29 ±0.37	nd	0.252 ±0.020	0.125 ±0.002	0.068 ±0.003	28.50 ±0.44	0.036 ±0.0004
17	10.1	*Taraxaci radix, Taraxacum officinale Weber ex Wiggers, Asteraceae*	6.08 ±0.52	11.70 ±0.60	8.49 ±0.55	0.033 ±0.002	0.073 ±0.006	0.532 ±0.020	0.082 ±0.005	4.84 ±0.48	0.082 ±0.005
18	10.2		4.68 ±0.19	10.53 ±0.91	6.40 ±0.35	nd	0.023 ±0.005	0.589 ±0.021	0.040 ±0.004	2.94 ±0.15	0.077 ±0.002
19	10.2		5.12 ±0.26	8.54 ±0.36	6.90 ±0.18	nd	0.015 ±0.001	0.537 ±0.014	0.054 ±0.003	3.27 ±0.23	0.054 ±0.001
20	11.1	*Galege herba, Galega officinalis* L., *Fabaceae*	3.84 ±0.23	23.24 ±1.01	15.70 ±0.64	0.024 ±0.008	0.351 ±0.023	0.227 ±0.011	0.048 ±0.007	8.36 ±0.47	0.061 ±0.002
21	11.2		5.25 ±0.12	11.94 ±0.96	12.76 ±0.20	0.085 ±0.009	0.516 ±0.025	0.218 ±0.010	0.049 ±0.001	9.09 ±1.16	0.136 ±0.006
22	12.1	*Silybi mariani fructus, Silybum marianum (L.) Gaertn., Asteraceae*	14.35 ±0.27	5.89 ±0.48	10.66 ±1.34	0.031 ±0.006	0.250 ±0.044	0.099 ±0.006	nd	5.05 ±0.56	0.021 ±0.002
23	12.2		13.82 ±0.23	5.15 ±0.10	12.93 ±0.31	0.040 ±0.004	0.464 ±0.024	0.102 ±0.003	nd	4.28 ±0.83	0.041 ±0.002
24	12.3		10.20 ±0.18	2.56 ±0.02	9.17 ±0.36	0.025 ±0.009	0.195 ±0.007	0.053 ±0.005	0.018 ±0.002	3.91 ±0.51	0.031 ±0.0005
25	13.1	*Graminis rhizoma, Agropyron repens (L.) P. Beauv, Poaceae*	1.39 ±0.30	7.47 ±0.52	2.98 ±0.12	nd	0.254 ±0.019	0.258 ±0.018	0.043 ±0.003	nd	0.017 ±0.0004
26	13.2		1.47 ±0.23	19.06 ±0.74	3.44 ±0.14	nd	0.041 ±0.006	0.311 ±0.012	0.044 ±0.002	nd	0.138 ±0.006
27	14.1	*Myrtilli folium, Vaccinium myrtillus* L., *Ericaceae*	2.24 ±0.20	598.20 ±20.88	14.51 ±0.06	nd	nd	0.138 ±0.006	0.028 ±0.003	9.93 ±0.64	0.026 ±0.001
28	14.2		2.69 ±0.26	498.79 ±11.93	12.01 ±0.21	nd	nd	0.137 ±0.0002	0.014 ±0.002	9.09 ±0.51	0.017 ±0.0004
29	15.1	*Salviae folium, Salvia officinalis* L., *Lamiaceae*	4.19 ±0.66	126.68 ±3.25	17.81 ±0.28	0.027 ±0.009	nd	0.188 ±0.010	0.018 ±0.001	7.14 ±0.52	0.086 ±0.003
30	15.2		3.31 ±0.41	42.99 ±0.55	9.24 ±0.54	0.048 ±0.010	nd	0.280 ±0.002	0.016 ±0.002	10.86 ±0.19	0.086 ±0.002
31	15.3		3.19 ±0.57	40.56 ±1.36	8.69 ±0.42	0.039 ±0.010	nd	0.259 ±0.013	0.008 ±0.001	8.92 ±0.37	0.055 ±0.002
32	16.1	*Menthae piperitae folium, Mentha x piperita* L., *Lamiaceae*	7.03 ±0.31	63.90 ±1.90	16.77 ±0.22	0.042 ±0.001	0.055 ±0.010	0.337 ±0.012	0.081 ±0.002	6.97 ±0.20	0.091 ±0.002
33	16.2		3.45 ±1.66	15.50 ±0.38	9.88 ±0.71	0.041 ±0.005	0.107 ±0.003	0.251 ±0.012	0.091 ±0.002	8.67 ±0.24	0.024 ±0.001
34	16.3		2.56 ±0.30	27.87 ±0.42	6.70 ±0.30	0.089 ±0.008	0.165 ±0.004	0.253 ±0.006	0.059 ±0.001	9.97 ±0.27	0.055 ±0.0004
35	17.1	*Lavandulae flos, Lavandula angustifolia Mill., Lamiaceae*	5.91 ±0.98	19.72 ±1.27	13.47 ±0.87	0.046 ±0.006	0.062 ±0.003	0.242 ±0.016	0.040 ±0.002	15.90 ±0.40	0.082 ±0.001
36	17.2		4.94 ±0.39	11.58 ±0.50	11.04 ±0.43	0.029 ±0.012	0.036 ±0.001	0.252 ±0.007	0.048 ±0.006	14.70 ±0.21	0.037 ±0.001
37	17.3		4.13 ±0.56	34.95 ±1.84	11.93 ±0.78	0.040 ±0.007	0.016 ±0.0004	0.246 ±0.010	0.038 ±0.003	20.32 ±0.96	0.100 ±0.006
38	18.1	*Ribis nigri folium, Ribes nigrum* L., *Grossulariaceae*	2.09 ±0.49	70.84 ±0.29	7.93 ±0.34	0.066 ±0.010	0.016 ±0.001	0.235 ±0.007	0.033 ±0.001	21.39 ±0.99	0.134 ±0.004
39	18.2		1.98 ±0.25	50.97 ±0.90	8.47 ±0.54	0.039 ±0.005	0.013 ±0.001	0.265 ±0.003	0.032 ±0.001	10.80 ±0.84	0.079 ±0.001
40	18.3		2.00 ±0.63	84.99 ±2.88	6.17 ±0.48	nd	nd	0.266 ±0.004	0.064 ±0.002	17.33 ±0.11	0.053 ±0.001
41	19.1	*Valerianae radix, Valeriana officinalis* L., *Valerianaceae*	6.68 ±0.79	9.40 ±0.39	6.05 ±0.18	nd	0.069 ±0.006	0.326 ±0.006	0.066 ±0.002	6.72 ±0.15	0.109 ±0.003
42	19.2		4.09 ±0.68	25.62 ±1.06	10.93 ±1.18	nd	0.014 ±0.004	0.249 ±0.012	0.031 ±0.002	2.99 ±0.38	0.127 ±0.008
43	20.1	*Chamomillae anthodium, Chamomilla recutita (L.) Rauschert, Asteraceae*	9.72 ±0.54	20.75 ±0.51	17.49 ±0.12	0.057 ±0.009	0.327 ±0.010	0.331 ±0.005	0.239 ±0.007	26.19 ±0.52	0.125 ±0.003
44	20.2		11.59 ±0.64	15.17 ±0.26	18.78 ±0.55	0.076 ±0.002	0.287 ±0.012	0.323 ±0.004	0.239 ±0.007	28.16 ±0.19	0.108 ±0.001
45	20.3		15.38 ±2.43	16.55 ±1.49	19.26 ±0.45	0.114 ±0.008	0.513 ±0.170	0.349 ±0.004	0.316 ±0.039	29.54 ±0.21	0.149 ±0.006
46	21.1	*Urticae folium, Urtica dioica* L., *Urtica urens* L., *Urticaceae*	7.30 ±0.67	9.58 ±0.37	20.88 ±1.42	0.052 ±0.011	1.053 ±0.068	0.207 ±0.016	0.070 ±0.006	16.63 ±1.23	0.045 ±0.003
47	21.2		3.70 ±0.31	11.76 ±0.36	18.02 ±0.66	0.027 ±0.001	0.802 ±0.163	0.183 ±0.004	0.058 ±0.002	24.92 ±0.91	0.030 ±0.0002
48	21.3		4.02 ±0.16	12.53 ±0.72	18.44 ±0.38	0.030 ±0.001	0.699 ±0.037	0.167 ±0.010	0.052 ±0.002	17.75 ±1.47	0.029 ±0.001
49	22.1	*Melissae folium, Melissa officinalis* L., *Lamiaceae*	6.11 ±0.56	18.21 ±0.41	12.26 ±0.23	0.084 ±0.011	0.112 ±0.004	0.330 ±0.012	0.069	15.37 ±0.19	0.055 ±0.002
									±0.003		
50	22.2		1.95 ±0.89	4.97 ±0.24	9.66 ±0.56	0.061 ±0.011	0.425 ±0.010	0.300 ±0.006	0.024 ±0.002	9.71 ±0.50	0.019 ±0.001
51	22.3		3.22 ±0.56	15.70 ±0.40	8.83 ±0.39	0.046 ±0.009	0.218 ±0.019	0.251 ±0.017	0.043 ±0.001	11.07 ±0.40	0.076 ±0.002
52	23.1	*Equiseti herba, Equisetum arvense L., Equisetaceae*	1.77 ±0.25	26.14 ±0.76	4.78 ±0.17	0.615 ±0.026	0.105 ±0.007	0.202 ±0.001	0.086 ±0.003	10.72 ±0.22	0.076 ±0.002
53	23.2		1.48 ±0.15	35.92 ±0.75	3.58 ±0.10	0.334 ±0.029	0.044 ±0.004	0.170 ±0.006	0.099 ±0.002	10.94 ±0.30	0.052 ±0.003
54	23.3		3.36 ±0.26	19.22 ±1.03	4.93 ±0.26	1.191 ±0.044	0.095 ±0.012	0.186 ±0.007	0.108 ±0.004	8.51 ±0.36	0.115 ±0.009

Products 1 to 34—Medicinal plants (*n* = 16) with indications for diabetes treatment (“MP-for-diabetes” group). Products 1–12 correspond to medicinal plants (*n* = 7) with a well-established therapeutic use in diabetes according to WHO monographs (first and second categories, i.e., “uses supported by clinical data” and “uses described in pharmacopeias and well-established documents”). Products 35–54—Medicinal plants (*n* = 7) with other therapeutic indications (“Other MP” group). The digit after the dot indicates different brands (marketing companies). All products were purchased in Gdansk, Poland, with the exception of products 4, 7, and (because these plants were not commercially available in Gdansk) 8–12 (which were purchased in Porto, Portugal). For more detailed information on brands/geographic origin, see Supplementary Materials, Table S1. nd = “not detected”, i.e., results below the limit of detection (LOD).

**Table 2:** Summary of the results (µg/g) for essential trace elements in medicinal plants as determined in their infusions (1 g sample: 100 mL boiling water) according to claimed therapeutic effect.

Element	Therapeutic Indication of the Plant	Mean	Standard Deviation	Median	95% Confidence Interval		*p*-Value
					Lower Limit	Upper Limit	
Mn *	Diabetes	51.5	125.6	10.5	9.6	93.3	0.041
	Other	25.7	21.0	18.7	15.9	35.5	
B *	Diabetes	11.7	10.0	8.7	8.4	15.0	0.008
	Other	16.0	7.3	15.6	12.5	19.4	
Zn	Diabetes	10.6	4.0	9.9	9.2	11.9	0.640
	Other	11.6	5.5	11.0	9.1	14.2	
Cu	Diabetes	4.75	2.96	4.19	3.76	5.74	0.960
	Other	5.07	3.64	4.05	3.37	6.78	
Cr	Diabetes	0.31	0.41	0.21	0.17	0.45	0.256
	Other	0.25	0.06	0.25	0.23	0.28	
Mo	Diabetes	0.30	0.63	0.11	0.09	0.51	0.894
	Other	0.25	0.30	0.10	0.10	0.39	
Co	Diabetes	0.07	0.06	0.06	0.05	0.09	0.349
	Other	0.08	0.04	0.08	0.06	0.10	
V *	Diabetes	0.05	0.03	0.04	0.04	0.06	0.041
	Other	0.09	0.08	0.06	0.05	0.13	
Se *	Diabetes	0.04	0.03	0.04	0.03	0.05	0.026
	Other	0.15	0.28	0.05	0.02	0.28	

* Statistically significant difference (*p* < 0.05) between both groups (plants used for diabetes vs. other medicinal plants).

**Table 3:** Summary of the results (µg/g) for toxic trace elements in medicinal plants as determined in their infusions (1 g sample: 100 mL boiling water) according to claimed therapeutic effect.

Element	Therapeutic Indication of the Plant	Mean	Standard Deviation	Median	95% Confidence Interval		*p*-Value
					Lower Limit	Upper Limit	
Ni	Diabetes	1.10	0.74	0.84	0.86	1.35	0.493
	Other	1.16	0.61	1.10	0.87	1.45	
As *	Diabetes	0.04	0.03	0.03	0.03	0.05	0.016
	Other	0.06	0.02	0.05	0.05	0.07	
Pb *	Diabetes	0.040	0.033	0.026	0.029	0.051	0.016
	Other	0.053	0.032	0.047	0.038	0.068	
Cd	Diabetes	0.016	0.020	0.009	0.009	0.022	0.841
	Other	0.010	0.010	0.007	0.006	0.015	
Tl	Diabetes	0.006	0.007	0.003	0.004	0.009	0.332
	Other	0.014	0.027	0.004	0.002	0.027	
Sb	Diabetes	0.0022	0.0018	0.0015	0.0016	0.0028	0.300
	Other	0.0022	0.0010	0.0021	0.0018	0.0027	
Be	Diabetes	0.0015	0.0016	0.0010	0.0009	0.0020	0.738
	Other	0.0016	0.0023	0.0009	0.0005	0.0027	

* Statistically significant difference (*p* < 0.05) between both groups (plants used for diabetes vs. other medicinal plants).

## Data Availability

Data is contained within the article.

## Supplementary Materials

The following supporting information can be downloaded at: https://www.mdpi.com/article/10.3390/foods11050667/s1, Table S1: Products’ brand (manufacturer)/geographic origin; Table S2: ICP-MS operating conditions; Table S3: Elemental isotopes (*m*/*z* ratios) monitored and limits of detection (LOD) of the ICP-MS method; Table S4: Levels (µg/g) of toxic trace elements in medicinal plants as determined in their infusions (1 g sample: 100 mL boiling water); Table S5: Summary of the results (µg/g) for other trace elements in medicinal plants as determined in their infusions (1 g sample: 100 mL boiling water) according to claimed therapeutic effect.
