# Supplementary material for: Trace Elements in Medicinal Plants Traditionally Used in the Treatment of Diabetes—Do They Have a Role in the Claimed Therapeutic Effect?
Source: Foods. 2022 Feb 24;11(5):667. doi: 10.3390/foods11050667 (PMC8909470; doi:10.3390/foods11050667)
Supplement: Supplementary file 1 [file foods-11-00667-s001.zip › foods-1561293-supplementary.pdf]

Supplementary Materials

Table S1. Products' brand (manufacturer) / geographic origin.

#Product	Plant . brand	Plant material, plant species and botanical family	Brand (manufacturer) / geographic origin
1	1.1	*Trigonellae foenugraeci semen, Trigonella foenum-graecum* L., *Fabaceae*	Kawon - PL
2	1.2		Farmwit - PL
3	1.3		Dary Natury - PL
4	1.4		Ervanário Portuense - **PT**
5	2.1	*Maydis stigma, Zea mays L., Poaceae (Gramineae)*	Kawon - PL
6	2.2		Flos - PL
7	2.3		Ervanário Portuense - **PT**
8	3.1	*Ocimi Sancti folium, Ocimum sanctum L., Lamiaceae*	BioSamara - **PT** (Origin: **Índia**)
9	4.1	*Azadirachti fructus, Azadirachta indica A. Juss., Meliaceae*	BioSamara - **PT** (Origin: **Índia**)
10	5.1	*Ginseng radix, Panax ginseng C.A. Meyer, Araliaceae*	Ervanário Portuense - **PT**
11	6.1	*Schisandrae fructus, Schisandra chinensis (Turcz.) Baill., Schisandraceae*	BioSamara - **PT** (Origin: **China**)
12	7.1	*Astragali radix, Astragalus membranaceus (Fisch.) Bunge,Fabaceae*	BioSamara - **PT** (Origin: **China**)
13	8.1	*Mori folium, Morus alba L., Moraceae*	Kawon - PL
14	8.2		Flos - PL
15	8.3		Dary Natury - PL
16	9.1	*Phaseoli pericarpium, Phaseolus vulgaris L., Fabaceae*	Kawon - PL
17	10.1	*Taraxaci radix, Taraxacum officinale Weber ex Wiggers, Asteraceae*	Kawon - PL
18	10.2		Flos - PL
19	10.2		Dary Natury - PL
20	11.1	*Galege herba, Galega officinalis L., Fabaceae*	Flos - PL
21	11.2		Farmwit - PL
22	12.1	*Silybi mariani fructus, Silybum marianum (L.) Gaertn., Asteraceae*	Farmwit - PL
23	12.2		Dary Natury - PL
24	12.3		Ol'Vita - PL
25	13.1	*Graminis rhizoma, Agropyron repens (L.) P. Beauv, Poaceae*	Kawon - PL
26	13.2		Dary Natury - PL
27	14.1	*Myrtilli folium, Vaccinium myrtillus* L.,*Ericaceae*	Flos - PL
28	14.2		Dary Natury - PL
29	15.1	*Salviae folium, Salvia officinalis L., Lamiaceae*	Kawon - PL
30	15.2		Flos - PL
31	15.3		Farmwit - PL
32	16.1	*Menthae piperitae folium, Mentha x piperita L., Lamiaceae*	Kawon - PL
33	16.2		Dary Natury - PL
34	16.3		Farmwit - PL
35	17.1	*Lavandulae flos, Lavandula angustifolia Mill., Lamiaceae*	Kawon - PL
36	17.2		Flos - PL
37	17.3		Dary Natury - PL
38	18.1	*Ribis nigri folium, Ribes nigrum L., Grossulariaceae*	Flos - PL
39	18.2		Dary Natury - PL
40	18.3		Farmwit - PL
41	19.1	*Valerianae radix, Valeriana officinalis L., Valerianaceae*	Kawon - PL
42	19.2		Dary Natury - PL
43	20.1	*Chamomillae anthodium, Chamomilla recutita (L.) Rauschert, Asteraceae*	Kawon - PL
44	20.1		Flos - PL
45	20.1		Dary Natury - PL
46	21.1	*Urticae folium, Urtica dioica L., Urtica urens L., Urticaceae*	Kawon - PL
47	21.2		Flos - PL
48	21.3		Dary Natury - PL
49	22.1	*Melissae folium, Melissa officinalis L., Lamiaceae*	Kawon - PL
50	22.2		Dary Natury - PL
51	22.3		Farmwit - PL
52	23.1	*Equiseti herba, Equisetum arvense L., Equisetaceae*	Kawon - PL
53	23.2		Dary Natury - PL
54	23.3		Farmwit - PL

Products 1 to 34 – Medicinal plants (**n=16**) with indication for diabetes treatment ("*MP-for-diabetes*" group). **Products 1-12** correspond to medicinal plants (**n=7**) with the well-established therapeutic use in diabetes according to WHO monographs (first and second categories, i.e., "uses supported by clinical data" and "uses described in pharmacopeias and well-established documents"). The digit after the dot indicates different brands (marketing companies). **Products 35 to 54** – Medicinal plants (**n=7**) with other therapeutic indication ("*Other MP*" group). **PT** – Portugal; **PL** – Poland. **Kawon** – located in Western Poland (Wielkopolska region). **Flos** – located in Western Poland (Wielkopolska region). **Farmwit** – located in Poland but registered in

the UK. **Dary Natury** – located in Eastern Poland (Podlaskie region). **Ol'Vita** – located in South-Western Poland (Dolnoslaskie region).

Table S2. ICP-MS operating conditions.

Parameter	Value
RF power (W)	1550
Cool flow (L/min)	14.0
Auxiliary argon flow rate (L/min)	0.8
Nebulizer flow rate (mL/min)	1.02
Spray chamber temperature (°C)	2.7
Number of replicates	3
Dwell time (ms)	0.05

Table S3. Elemental isotopes (m/z ratios) monitored and Limits of Detection (LOD) of the ICP-MS method.

Elemental isotope	LOD (µg/g)
^{7}Li	0.002
^{9}Be	0.0002
^{11}B	1.402
^{27}Al	0.285
^{45}Sc	0.010
^{51}V	0.003
^{52}Cr	0.011
^{55}Mn	0.092
^{59}Co	0.0004
^{60}Ni	0.010
^{65}Cu	0.042
^{66}Zn	0.194
^{75}As	0.027
^{82}Se	0.022
^{85}Rb	0.010
^{88}Sr	0.010
^{89}Y	0.0004
^{98}Mo	0.009
^{111}Cd	0.0006
^{121}Sb	0.0005
^{137}Ba	0.092
^{141}Pr	0.00008
^{146}Nd	0.0003
^{147}Sm	0.0002
^{153}Eu	0.00008
^{157}Gd	0.0002
^{159}Tb	0.00006
^{163}Dy	0.00008
^{165}Ho	0.00007
^{166}Er	0.0002
^{169}Tm	0.00005
^{172}Yb	0.00012
^{175}Lu	0.0001
^{205}Tl	0.00010
^{208}Pb	0.007
^{209}Bi	0.0015
^{238}U	0.0002
^{71}Ga (IS)	
^{115}In (IS)	
^{195}Pt (IS)	

IS – Internal Standard

Table S4. Levels (μg/g) of **toxic trace elements** in medicinal plants as determined in their infusions (1 g sample : 100 ml boiling water).

Prod	Pl/Br	Plant name	Be	Cd	Tl	Pb	As	Sb	Ni
1	1.1	*Trigonellae foenugraeci semen*	nd	0.0180 ±0.0007	0.0014 ±0.0000	0.0175 ±0.0112	nd	nd	3.078 ±0.055
2	1.2		nd	0.0072 ±0.0006	0.0018 ±0.0001	0.0293 ±0.0101	0.0256 ±0.0120	nd	1.314 ±0.049
3	1.3		nd	0.0086 ±0.0004	0.0006 ±0.0001	0.0158 ±0.0105	nd	nd	1.173 ±0.053
4	1.4		nd	0.0100 ±0.0004	0.0007 ±0.0000	0.0112 ±0.0087	nd	nd	0.635 ±0.026
5	2.1	*Maydis stigma*	nd	0.0011 ±0.0002	0.0005 ±0.0001	0.0141 ±0.0010	0.0415 ±0.0037	0.0010 ±0.0002	0.473 ±0.030
6	2.2		0.0003 ±0.0001	0.0011 ±0.0002	0.0004 ±0.0001	0.0167 ±0.0017	0.0437 ±0.0031	0.0014 ±0.0001	0.636 ±0.023
7	2.3		nd	0.0006 ±0.0002	0.0002 ±0.0001	0.0143 ±0.0067	nd	0.0022 ±0.0013	0.241 ±0.059
8	3.1	*Ocimi Sancti folium*	0.0018 ±0.0003	0.0037 ±0.0005	0.0034 ±0.0002	0.0671 ±0.0116	0.0985 ±0.0097	0.0009 ±0.0003	0.840 ±0.021
9	4.1	*Azadirachti fructus*	nd	0.0041 ±0.0003	0.0034 ±0.0000	0.0774 ±0.0203	0.0336 ±0.0016	0.0007 ±0.0000	0.388 ±0.027
10	5.1	*Ginseng radix*	0.0045 ±0.0006	0.0288 ±0.0012	0.0222 ±0.0005	0.0837 ±0.0159	0.0582 ±0.0068	0.0046 ±0.0007	0.644 ±0.016
11	6.1	*Schisandrae fructus*	0.0047 ±0.0001	0.0190 ±0.0005	0.0025 ±0.0001	0.1362 ±0.0209	0.0853 ±0.0055	0.0064 ±0.0010	2.241 ±0.044
12	7.1	*Astragali radix*	0.0034 ±0.0018	0.0097 ±0.0018	0.0093 ±0.0001	0.0164 ±0.0041	0.0895 ±0.0065	0.0073 ±0.0010	0.821 ±0.015
13	8.1	*Mori folium*	nd	0.0013 ±0.0004	0.0004 ±0.0001	0.0576 ±0.0059	0.0740 ±0.0065	0.0024 ±0.0003	1.628 ±0.0619
14	8.2		nd	0.0031 ±0.0002	0.0006 ±0.0000	0.1315 ±0.0119	0.1110 ±0.0031	0.0035 ±0.0001	1.609 ±0.016
15	8.3		nd	0.0022 ±0.0003	0.0004 ±0.0000	0.0747 ±0.0164	0.1272 ±0.0060	0.0060 ±0.0024	1.448 ±0.038
16	9.1	*Phaseoli pericarpium*	0.0018 ±0.0003	0.0034 ±0.0005	0.0034 ±0.0001	0.0208 ±0.0097	0.0328 ±0.0083	0.0024 ±0.0003	0.750 ±0.052
17	10.1	*Taraxaci radix*	0.0018 ±0.0001	0.0497 ±0.0028	0.0122 ±0.0008	0.0156 ±0.0062	0.0366 ±0.0100	0.0021 ±0.0006	2.534 ±0.182
18	10.2		0.0017 ±0.0004	0.0442 ±0.0034	0.0113 ±0.0005	0.0103 ±0.0011	nd	0.0010 ±0.0002	2.594 ±0.068
19	10.2		0.0022 ±0.0003	0.0357 ±0.0017	0.0055 ±0.0003	0.0165 ±0.0049	nd	0.0020 ±0.0001	2.072 ±0.073
20	11.1	*Galege herba*	nd	0.0090 ±0.0005	0.0147 ±0.0007	0.0432 ±0.0200	nd	0.0013 ±0.0002	0.557 ±0.013
21	11.2		nd	0.0030 ±0.0004	0.0028 ±0.0000	0.0235 ±0.0059	nd	0.0024 ±0.0007	2.158 ±0.071
22	12.1	*Silybi mariani fructus*	nd	0.0716 ±0.0074	0.0148 ±0.0002	0.0206 ±0.0019	nd	nd	1.096 ±0.029
23	12.2		nd	0.0205 ±0.0019	0.0152 ±0.0000	0.0240 ±0.0070	nd	nd	0.486 ±0.004
24	12.3		0.0010 ±0.0002	0.0857 ±0.0014	0.0011 ±0.0001	0.0119 ±0.0065	nd	0.0012 ±0.0003	0.841 ±0.008
25	13.1	*Graminis rhizoma*	0.0013 ±0.0001	0.0099 ±0.0007	0.0081 ±0.0004	0.0299 ±0.0109	nd	0.0030 ±0.0003	0.114 ±0.008
26	13.2		0.0036 ±0.0003	0.0219 ±0.0007	0.0129 ±0.0010	0.0306 ±0.0071	0.0508 ±0.0079	0.0033 ±0.0004	0.158 ±0.008
27	14.1	*Myrtilli folium*	0.0014 ±0.0003	0.0260 ±0.0008	0.0225 ±0.0012	0.0173 ±0.0050	0.0960 ±0.0097	0.0012 ±0.0001	0.492 ±0.009
28	14.2		0.0021 ±0.0002	0.0129 ±0.0004	0.0092 ±0.0001	0.0204 ±0.0066	0.0350 ±0.0026	0.0015 ±0.0003	0.740 ±0.022

Table S4. Levels (µg/g) of **toxic trace elements** in medicinal plants as determined in their infusions (1 g sample : 100 ml boiling water). (cont.)

Prod	Pl/Br	Plant name	Be	Cd	Tl	Pb	As	Sb	Ni
29	15.1	*Salviae folium*	0.0015 ±0.0001	0.0048 ±0.0002	0.0059 ±0.0001	0.0676 ±0.0154	nd	0.0007 ±0.0004	0.947 ±0.005
30	15.2		0.0013 ±0.0001	0.0023 ±0.0002	0.0036 ±0.0000	0.0294 ±0.0090	nd	nd	0.622 ±0.008
31	15.3		0.0007 ±0.0002	0.0012 ±0.0002	0.0018 ±0.0001	0.0257 ±0.0105	nd	0.0013 ±0.0016	0.774 ±0.021
32	16.1	*Menthae piperitae folium*	0.0009 ±0.0001	0.0040 ±0.0006	0.0023 ±0.0001	0.1010 ±0.0177	0.0660 ±0.0046	0.0007 ±0.0003	1.235 ±0.020
33	16.2		0.0005 ±0.0002	0.0018 ±0.0002	0.0022 ±0.0001	0.0605 ±0.0438	0.0588 ±0.0043	0.0007 ±0.0001	0.222 ±0.011
34	16.3		0.0006 ±0.0001	0.0129 ±0.0002	0.0025 ±0.0001	0.0488 ±0.0055	0.0529 ±0.0040	0.0009 ±0.0001	1.239 ±0.057
35	17.1	*Lavandulae flos*	0.0009 ±0.0002	0.0026 ±0.0002	0.0024 ±0.0000	0.0520 ±0.0181	0.0510 ±0.0078	0.0036 ±0.0001	1.368 ±0.047
36	17.2		0.0006 ±0.0002	0.0025 ±0.0003	0.0023 ±0.0000	0.0366 ±0.0098	0.0328 ±0.0072	0.0015 ±0.0000	1.097 ±0.099
37	17.3		0.0013 ±0.0007	0.0057 ±0.0009	0.0029 ±0.0002	0.0675 ±0.0156	0.0375 ±0.0042	0.0021 ±0.0006	1.096 ±0.075
38	18.1	*Ribis nigri folium*	0.0011 ±0.0004	0.0024 ±0.0002	0.0034 ±0.0000	0.0338 ±0.0138	0.0415 ±0.0046	0.0009 ±0.0002	1.716 ±0.025
39	18.2		0.0010 ±0.0002	0.0067 ±0.0008	0.0038 ±0.0002	0.0425 0.0042	0.0492 ±0.0073	0.0016 ±0.0001	2.006 ±0.104
40	18.3		0.0020 ±0.0001	0.0290 ±0.0017	0.0052 ±0.0002	0.0484 ±0.0127	0.0467 ±0.0019	0.0023 ±0.0003	1.247 ±0.016
41	19.1	*Valerianae radix*	0.0018 ±0.0000	0.0081 ±0.0001	0.0261 ±0.0012	0.0506 ±0.0111	0.0822 ±0.0062	0.0031 ±0.0004	0.724 ±0.032
42	19.2		0.0050 ±0.0003	0.0399 ±0.0043	0.0312 ±0.0004	0.0486 ±0.0119	0.0338 ±0.0013	0.0031 ±0.0003	1.472 ±0.051
43	20.1	*Chamomillae anthodium*	0.0005 ±0.0000	0.0092 ±0.0004	0.0011 ±0.0001	0.0347 ±0.0066	0.0678 ±0.0073	0.0021 ±0.0001	2.077 ±0.016
44	20.2		0.0003 ±0.0001	0.0102 ±0.0006	0.0008 ±0.0000	0.0324 ±0.0111	0.0792 ±0.0083	0.0020 ±0.0004	1.862 ±0.009
45	20.3		0.0103 ±0.0079	0.0212 ±0.0066	0.0025 ±0.0014	0.0673 ±0.0507	0.1053 ±0.0185	0.0044 ±0.0021	2.298 ±0.027
46	21.1	*Urticae folium*	0.0022 ±0.0007	0.0078 ±0.0018	0.0149 ±0.0010	0.0642 ±0.0124	0.0841 ±0.0177	0.0035 ±0.0013	0.873 ±0.049
47	21.2		0.0008 ±0.0001	0.0053 ±0.0006	0.1191 ±0.0056	0.0253 ±0.0082	0.0638 ±0.0064	0.0019 ±0.0000	0.323 ±0.004
48	21.3		0.0007 ±0.0002	0.0046 ±0.0004	0.0404 ±0.0004	0.0392 ±0.0079	0.0505 ±0.0039	0.0022 ±0.0001	0.398 ±0.009
49	22.1	*Melissae folium*	0.0003 ±0.0002	0.0016 ±0.0002	0.0021 ±0.0000	0.0495 ±0.0088	0.0708 ±0.0060	0.0011 ±0.0002	1.412 ±0.014
50	22.2		nd	0.0140 ±0.0179	0.0020 ±0.0001	0.1757 ±0.2184	0.0289 ±0.0104	0.0016 ±0.0002	0.151 ±0.006
51	22.3		0.0004 ±0.0002	0.0050 ±0.0004	0.0038 ±0.0001	0.0726 ±0.0101	0.0298 ±0.0016	0.0013 ±0.0001	1.025 ±0.021
52	23.1	*Equiseti herba*	0.0005 ±0.0001	0.0175 ±0.0096	0.0118 ±0.0005	0.0461 ±0.0378	0.0795 ±0.0095	0.0024 ±0.0001	0.835 ±0.016
53	23.2		0.0010 ±0.0000	0.0085 ±0.0040	0.0058 ±0.0001	0.0328 ±0.0301	0.0559 ±0.0035	0.0007 ±0.0002	0.581 ±0.023
54	23.3		0.0005 ±0.0001	0.0056 ±0.0002	0.0029 ±0.0002	0.0376 ±0.0029	0.1100 ±0.0046	0.0033 ±0.0007	0.646 ±0.010

nd = "not detected", i.e., results below the limit of detection (LOD). Prod = Product; Pl/Br = Plant / Brand.

Table S5. Summary of the results (μg/g) for **other trace elements** in medicinal plants as determined in their infusions (1 g sample : 100 ml boiling water) according to claimed therapeutic effect.

Element	Therapeutic indication of the plant	Mean	Standard deviation	Median	95% confidence interval	
					lower limit	upper limit
**Li**	Diabetes	0.11	0.16	0.06	0.06	0.17
	Other	0.09	0.11	0.05	0.04	0.14
**Sr***	Diabetes	11.39	25.90	**4.63**	2.76	20.03
	Other	15.39	11.57	15.05	9.98	20.81
Al	Diabetes	6.78	14.17	1.38	2.06	11.51
	Other	3.71	3.78	3.15	1.95	5.48
Rb	Diabetes	8.55	5.37	6.75	6.76	10.34
	Other	13.21	14.36	8.82	6.49	19.93
Ba	Diabetes	3.63	5.13	1.87	1.92	5.34
	Other	4.50	4.22	3.55	2.52	6.47
**Sc***	Diabetes	0.07	0.04	**0.06**	0.06	0.09
	Other	0.15	0.13	0.10	0.09	0.21
Bi	Diabetes	0.052	0.045	0.034	0.037	0.067
	Other	0.066	0.029	0.069	0.052	0.080
Y	Diabetes	0.007	0.009	0.004	0.004	0.009
	Other	0.007	0.007	0.005	0.003	0.010
Nd	Diabetes	0.0051	0.0061	0.0025	0.0030	0.0071
	Other	0.0043	0.0049	0.0028	0.0020	0.0066
Er	Diabetes	0.0016	0.0011	0.0012	0.0012	0.0019
	Other	0.0013	0.0007	0.0012	0.0010	0.0016
Pr	Diabetes	0.0014	0.0017	0.0007	0.0009	0.0020
	Other	0.0012	0.0012	0.0009	0.0007	0.0018
Sm	Diabetes	0.0013	0.0013	0.0007	0.0009	0.0017
	Other	0.0011	0.0012	0.0008	0.0006	0.0017
Gd	Diabetes	0.0012	0.0013	0.0007	0.0008	0.0017
	Other	0.0012	0.0013	0.0009	0.0006	0.0018
Dy	Diabetes	0.0012	0.0013	0.0006	0.0008	0.0016
	Other	0.0010	0.0011	0.0008	0.0005	0.0015
Eu	Diabetes	0.0010	0.0010	0.0008	0.0007	0.0013
	Other	0.0012	0.0008	0.0009	0.0008	0.0016
Yb	Diabetes	0.0007	0.0009	0.0004	0.0004	0.0010
	Other	0.0005	0.0005	0.0005	0.0003	0.0007
**Tb***	Diabetes	0.0006	0.0005	**0.0006**	0.0004	0.0007
	Other	0.0012	0.0008	0.0007	0.0008	0.0015
Ho	Diabetes	0.0005	0.0009	0.0001	0.0002	0.0008
	Other	0.0003	0.0003	0.0002	0.0002	0.0005
Tm	Diabetes	0.0004	0.0009	0.0000	0.0001	0.0007
	Other	0.0002	0.0002	0.0001	0.0001	0.0003
Lu	Diabetes	0.0004	0.0009	0.0001	0.0001	0.0007
	Other	0.0002	0.0002	0.0002	0.0001	0.0004

*Statistically significant difference ($p < 0.05$) between both groups (plants used for diabetes vs. other medicinal plants).
